# Architectural and Biochemical Expressions of Mustard Gas Keratopathy: Preclinical Indicators and Pathogenic Mechanisms

**DOI:** 10.1371/journal.pone.0042837

**Published:** 2012-08-10

**Authors:** Patrick McNutt, Megan Lyman, Adam Swartz, Kaylie Tuznik, Denise Kniffin, Kim Whitten, Denise Milhorn, Tracey Hamilton

**Affiliations:** 1 United States Army Medical Research Institute of Chemical Defense, Aberdeen Proving Ground, Maryland, United States of America; 2 United States Army Medical Research and Materiel Command, Fort Detrick, Maryland, United States of America; Wayne State University, United States of America

## Abstract

A subset of victims of ocular sulfur mustard (SM) exposure develops an irreversible, idiotypic keratitis with associated secondary pathologies, collectively referred to as mustard gas keratopathy (MGK). MGK involves a progressive corneal degeneration resulting in chronic ocular discomfort and impaired vision for which clinical interventions have typically had poor outcomes. Using a rabbit corneal vapor exposure model, we previously demonstrated a clinical progression with acute and chronic sequelae similar to that observed in human casualties. However, a better understanding of the temporal changes that occur during the biphasic SM injury is crucial to mechanistic understanding and therapeutic development. Here we evaluate the histopathologic, biochemical and ultrastructural expressions of pathogenesis of the chronic SM injury over eight weeks. We confirm that MGK onset exhibits a biphasic trajectory involving corneal surface regeneration over the first two weeks, followed by the rapid development and progressive degeneration of corneal structure. Preclinical markers of corneal dysfunction were identified, including destabilization of the basal corneal epithelium, basement membrane zone abnormalities and stromal deformation. Clinical sequelae of MGK appeared abruptly three weeks after exposure, and included profound anterior edema, recurring corneal erosions, basement membrane disorganization, basal cell necrosis and stromal degeneration. Unlike resolved corneas, MGK corneas exhibited frustrated corneal wound repair, with significantly elevated histopathology scores. Increased lacrimation, disruption of the basement membrane and accumulation of pro-inflammatory mediators in the aqueous humor provide several mechanisms for corneal degeneration. These data suggest that the chronic injury is fundamentally distinct from the acute lesion, involving injury mechanisms that operate on different time scales and in different corneal tissues. Corneal edema appears to be the principal pathology of MGK, in part resulting from persistent necrosis of the basal corneal epithelium and deterioration of the basement membrane. The findings also provide a potential explanation as to why administration of anti-inflammatories transiently delays, but does not prevent, the development of MGK sequelae.

## Introduction

Sulfur mustard (2,2′-dichloroethylsulfide; SM) is a highly reactive, alkylating chemical that causes severe clinical morbidities following topical or inhalational exposures. Because of the relatively high sensitivity of corneal tissues, even low levels of ocular SM exposure can result in debilitating injuries [1]. Battlefield deployment of SM as a chemical weapon in British trenches during WWI and in the Iran-Iraq war resulted in over 210,000 casualties, 90% of whom presented with acute ocular SM injuries [2]. Unlike ocular exposure to other chemical agents, in which victims undergo injury resolution, a subset of victims of SM exposure subsequently developed chronic corneal symptoms that required clinical management decades after exposure [3], [4], [5], [6], [7], [8]. Patients developed corneal pathologies, such as chronic keratitis, persistent corneal erosions and neovascularization, either immediately after exposure or following a clinically asymptomatic period of 0.5 to 40 years [6]. Together, these symptoms comprise the pathophysiologic condition termed mustard gas keratopathy (MGK).

In contrast to the acute epithelial lesion, which resolves within weeks, MGK appears to involve a slow progression towards destructive inflammation, resulting in corneal degeneration that can cause a permanent reduction in visual acuity or complete loss of eyesight [6]. The pathogenesis of MGK has been clinically described as a chronic keratitis with secondary keratopathies, such as chronic epithelial lesions, corneal neovascularization and progressive corneal degeneration [5], [6], [7]. Despite recent progress in understanding how the cornea responds to acute insults, the unknown etiology of MGK has contributed to the failure of therapeutic approaches, limiting treatment to palliative measures applied succedent to the onset of symptoms [5], [9].

Human and rabbit corneas are structurally similar, and rabbit corneas exposed to SM vapor exhibit many of the same acute and chronic symptoms as have been described in human victims [1], [5], [6], [10]. We previously used the rabbit vapor exposure model to characterize the histopathology of the acute injury and identify clinical metrics of MGK onset [11], [12]. Within 1 d of corneal SM administration, exposed corneal epithelium (CE) sloughs from the basement membrane (BM), corneal edema develops in the denuded stroma and full-thickness keratocytosis is apparent within the wound margins. By five days, an epithelial cap is regenerated and corneal edema begins to subside. One week after exposure, the CE is partially stratified, with rudimentary hemidesmosomal attachments. Despite this apparent improvement, corneas develop clinical signatures of chronic injury as soon as three weeks after exposure, including persistently elevated corneal edema, recurring corneal erosions and neovascularization. By eight weeks, the basement membrane zone (BMZ) had undergone severe degeneration. The structural changes responsible for the transition from corneal recovery at one week to corneal degeneration at eight weeks are unknown. Those corneas that did not develop chronic injury (resolved corneas) become clinically asymptomatic by five weeks.

Steroidal anti-inflammatory therapies delay but do not prevent MGK, suggesting that the underlying mechanisms of chronic SM toxicity are refractory to steroidal treatment [13], [14]. Here we used histopathology, transmission electron microscopy (TEM) and biochemistry to evaluate pathologic expressions of the biphasic injury and develop an improved understanding of the biological processes involved in the appearance and progression of the chronic phase sequelae. We applied these approaches to evaluate temporal changes in corneal structure from 1–8 weeks and correlated changes to clinical indications; characterized MGK-related degeneration of the basement membrane zone (BMZ) and stroma; compared wound healing processes between resolving and MGK corneas; and identified early sequelae associated with MGK onset. We also evaluated whether MGK corneas experience increased exposure to keratoactive substances. An improved understanding of the temporal changes in corneal structure and biochemistry associated with MGK is essential to mechanistic understanding of pathogenesis and identification of viable therapeutic approaches.

## Materials and Methods

### Ethics Statement and Disclaimers

The experimental protocol was approved by the Animal Care and Use Committee at the United States Army Medical Research Institute of Chemical Defense (USDA certificate number 51-F-0006). All procedures were conducted in accordance with the principles stated in the Guide for the Care and Use of Laboratory Animals and the Animal Welfare Act of 1966 (P.L. 89–544), as amended. The views expressed in this manuscript are those of the author(s) and do not reflect the official policy of the Department of Army, Department of Defense, or the U.S. Government.

### Animals

Forty-eight female New Zealand white rabbits (Charles River Laboratories) weighing 2.0–2.5 kilograms were housed individually in approved cages. Rabbits were provided a standard diet with regular enrichment and water *ad libitum*.

### Exposure Procedures

Rabbits were exposed in 16 animal cohorts at 8-week intervals. One day prior to exposure a 2 in by 2 in region on the rabbit’s back was clipped and a 25 µg/hr fentanyl patch was placed anterior to the scapula [15]. On the day of exposure, rabbits were anesthetized with an intramuscular administration of 15 mg/kg of ketamine and 7 mg/kg of xylazine and physiological parameters recorded. The right corneas of anesthetized rabbits were exposed to SM vapor for 2.5 min using a vapor cup delivery system as previously described [11], [12]. Two min after exposure, exposed eyes were flushed with 10 mL of sterile saline to remove residual agent. Rabbits were returned to cages and provided food and water *ad libitum*. Serial application of fentanyl patches was used to ensure rabbits received pain management through 6 d after SM exposure. Animals were monitored daily for signs of pain and distress. Corneal injury was clinically evaluated on a weekly basis using measurements of corneal thickness, fluorescein exclusion assays and white light and slit-lamp images of the cornea.

### Histologic Examination

Corneas that underwent a significant decrease in corneal edema at three weeks and did not develop persistent epithelial erosions were considered to be resolving, and were harvested for histopathology at eight weeks. Otherwise, corneas were randomly selected for analysis at each time point. For corneal harvest, rabbits were euthanized in groups of four at 1 day, weekly from 1–5 and at 8 weeks. Enucleated rabbit eyes were fixed by injection of 1.6% buffered paraformaldehyde and 2.5% glutaraldehyde into the posterior chamber, followed by immersion in the same fixative at 4°C for 24–28 h. Corneas were dissected from the globe through the limbus, and representative areas of tissue to include any gross lesions were isolated by scalpel and processed for routine hematoxylin and eosin (H&E) light microscopy and transmission electron microscopy. For light microscopy, fixed corneas from all animals were processed, embedded in paraffin wax, sectioned (5 µm) on a rotary microtome, mounted on glass slides, and stained with H&E. Light microscopy was performed using an Olympus BX51 microscope. The degree and extent of epithelial and stromal sequelae in sagittal cross-sections were graded as previously described, to include epithelial attenuation and necrosis and stromal necrosis, deformation, neovascularization and inflammation [12]. Exposed corneas were systematically classified and scored by a veterinary pathologist in the following manner: 0 indicates no evidence of injury; 1 indicates injury is present in 1–10% of each cornea; 2 indicates injury is observed in 11–25% of each cornea; 3 indicates injury is observed in 26–50% of each cornea; and 4 indicates injury is distributed in over 50% of the each cornea.

### Transmission Electron Microscopy (TEM)

For one- and two-week samples, corneas were selected for TEM at random; beyond two weeks corneas were selected only if they exhibited characteristic clinical evidence of MGK [11]. Following enucleation and fixation as above, selected corneas were post-fixed in buffered 1% osmium tetroxide, dehydrated in graded ethanol and embedded in Poly/Bed® 812 resin. Ninety-nanometer thick sections were mounted on copper mesh grids and counter-stained with uranyl acetate and lead citrate. TEM was performed using a JEOL JEM-1230 transmission electron microscope. For TEM, observations of sequelae in at least three corneas were considered genuine. Fixation techniques optimized to preserve the structures of the stroma and epithelium proved to be suboptimal for endothelial cell preservation.

### Measurement of Inflammatory Mediators in the Aqueous Humor

Matched paracentesis samples and plasma were longitudinally collected at 0, 1 and 7 weeks after SM exposure (n = 6 per condition) and stored. For paracentesis, animals were anesthetized (7.5 mg/kg ketamine and 3.5 mg/kg xylazine i.m.), and anterior chamber aqueous humor (AH) was obtained under aseptic conditions using a 30 gauge ½” sterile needle. AH was transferred to a cryo-vial and frozen at −20°C. Plasma was collected from venous bleeds as previously described [11]. Protein concentrations were determined by bicinchoninic acid protein assay (Pierce, Rockford, IL). AH and plasma were assayed for interleukin-1β (IL-1β), tumor necrosis factor-α (TNF-α) IL-6, IL-8, GM-CSF, IFN-γ, IL-10, IL-12p70 and IL-2 protein using a Meso Scale Discovery SI2400 ECL imager according to manufacturer’s protocol (Meso Scale Discovery, Gaithersburg, MD). Activated matrix metalloproteinase-2 (MMP-2) and -9 in AH were quantified by zymography on pre-cast 10% gelatin zymogram gels (Invitrogen, Carlsbad, CA) per manufacturer’s protocols and densitometry images were normalized to total protein.

### Schirmer Tear Test (STT)

Longitudinal changes in tear production were evaluated from 0–5 weeks using the STT (n = 8). Sterile STT papers (Fisher Scientific, Pittsburgh, PA) were inserted within the lower conjunctival fornix near the junction of the medio-temporal third of the right eyelid. After 60 s the test paper was removed, and the degree of wetting was measured immediately with a mechanical ruler. Control corneas produced tear quantities similar to those previously reported in rabbits [16].

### Statistical Comparisons

Multiple comparisons of histology scores were performed using Kruskal-Wallis one-way analysis of variance (ANOVA), and significances were determined by comparison to two-week means using Dunn’s multiple comparisons test. Two-week scores were used as a baseline, since these represent the point at which the cornea exhibits the lowest, non-zero injury scores. Longitudinal changes in lacrimation, cytokine and MMP levels were compared using repeated-measures ANOVA, and significances were determined using the Tukey-Kramer multiple comparisons test. Significances are provided in figure legends.

## Results

### Histopathology from 1–8 Weeks Confirms Clinical Evidence for a Biphasic SM Ocular Injury

**Figure 1:**
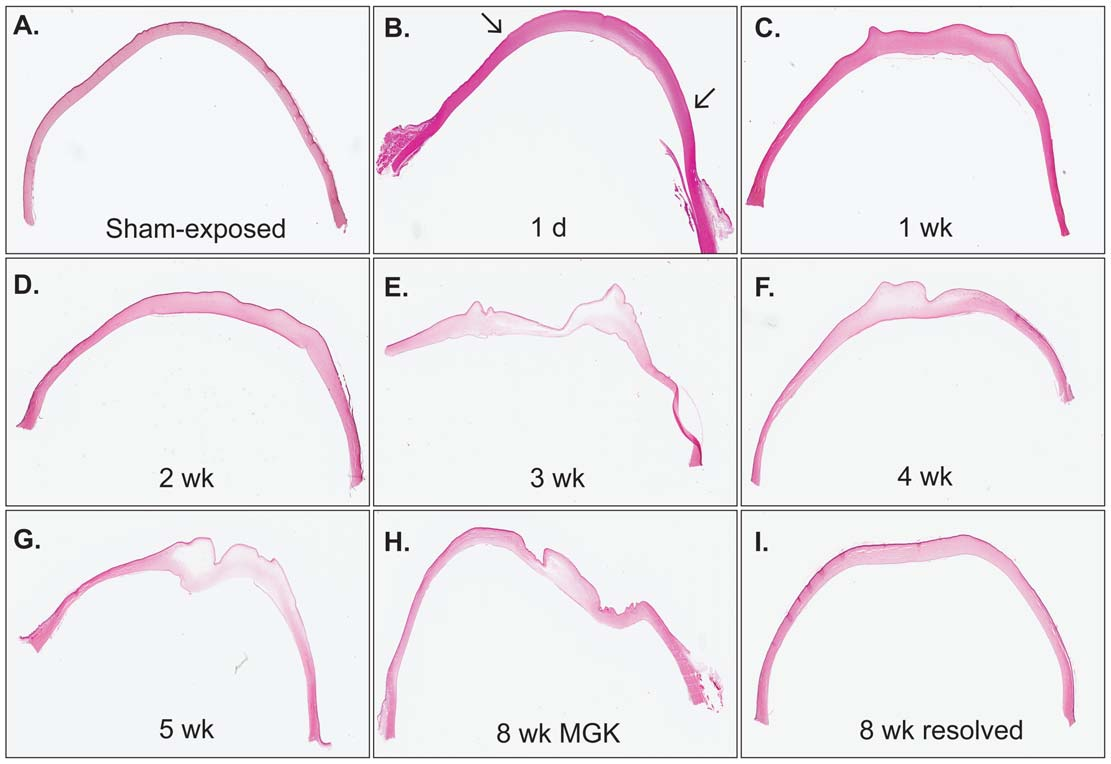
Light microscopy of H&E-stained sections demonstrating representative changes in corneal histology from 1–8 weeks after a 2.5-min SM vapor exposure. Arrows in panel B represent boundaries of acute epithelial lesion.

Clinical metrics of injury progression in exposed corneas were similar to those previously reported, with 90% of corneas developing a chronic injury [11]. Similar to previous findings, SM exposure caused detachment and sloughing of the exposed CE within 24 h, accompanied by stromal edema beneath the lesion. Here we report that over the first two weeks following exposure, corneas re-epithelialized and began to undergo a reduction in corneal edema, suggestive of corneal healing from the acute SM lesion (Figure 1A–D). Corneal histology deteriorated abruptly between two and three weeks with the development of epithelial lesions; epithelial and stromal edema; neovascularization; stromal deformity; and stromal loss (Figure 1E–H). Conversely, resolved corneas exhibited minimal evidence of injury at eight weeks.

**Figure 2:**
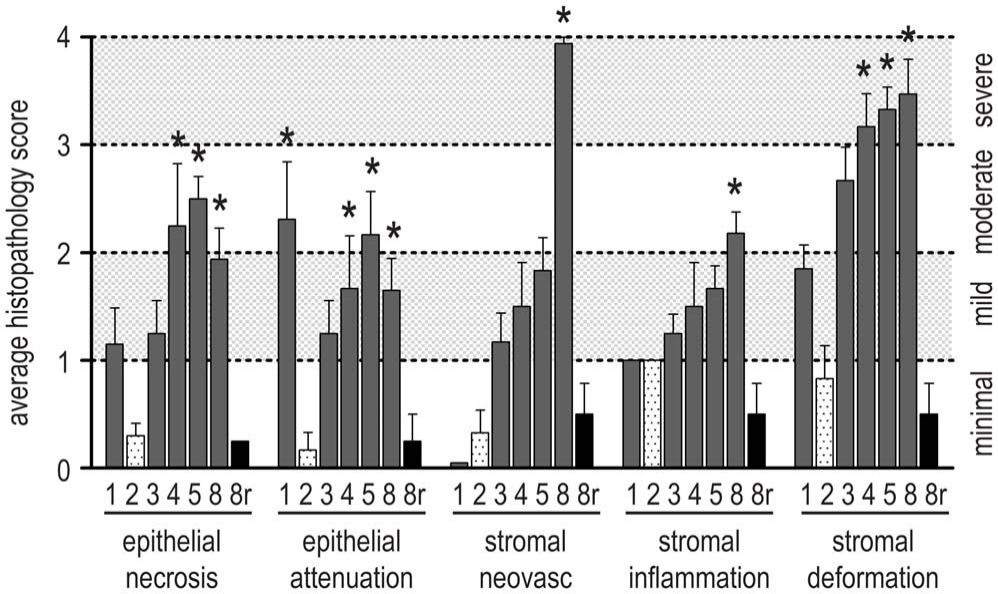
Histopathology scores over an 8 week period following a 2.5-min corneal exposure to SM vapor. The degree and extent of corneal sequelae from 1–8 weeks (n = 6 for all) and resolved cornea at 8 weeks (8r; n = 4; black bars) were scored. Qualitative descriptors corresponding to numerical scores are presented on the right axis. Data shown represent mean plus standard error and asterisks indicate values that are significantly different from 2-week scores (*p*<0.05; cross-hatched bars).

Histopathologic scoring of corneas corroborated evidence of a biphasic injury (Figure 2). The significant improvement in the acute injury between one and two weeks was followed by a progressive deterioration in MGK corneas that resulted in moderate or severe scores in most categories at eight weeks. Notably, resolving corneas showed no significant differences between two and eight weeks.

The limbus and conjunctiva did not display symptoms of injury nor was goblet cell hyperplasia observed in the cornea. Acute or chronic sequelae were not observed in sham-exposed corneas.

### MGK Corneas Exhibit Delays in Wound Repair Processes

**Figure 3:**
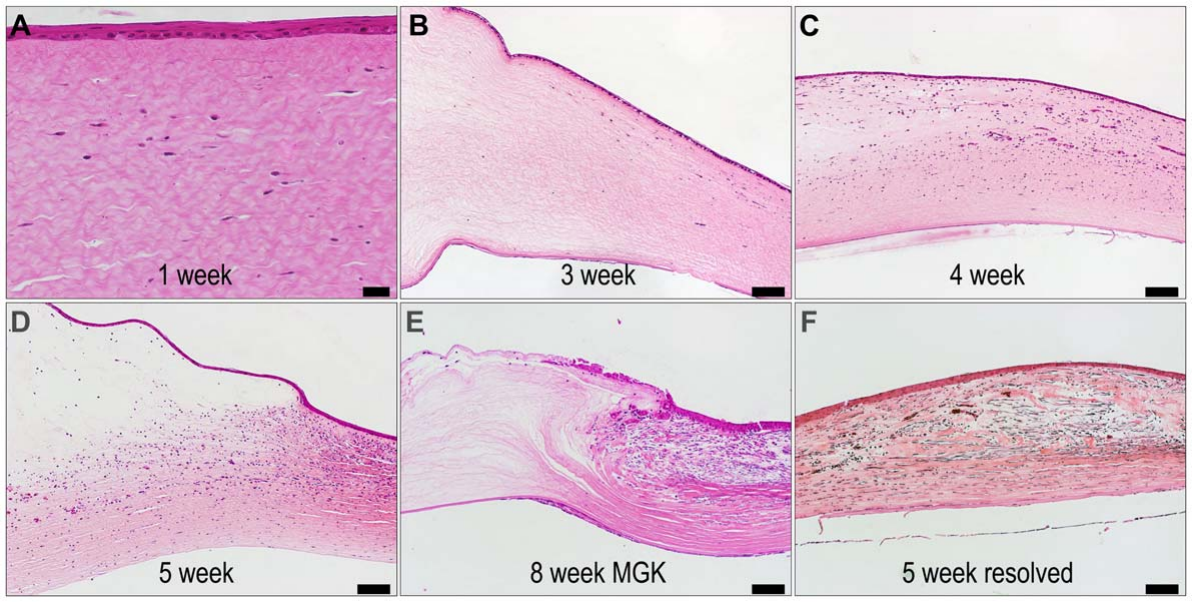
Representative light microscopy of H&E-stained sections demonstrating cellular aspects of wound repair in MGK and resolving corneas. Panels A–D were imaged at the lesion margin, with the limbus to the right, and panels E–F were imaged at the central cornea. Scale bar is 100 µm for all panels except 1 week, where it is 20 µm.

Corneas that developed chronic injury were characterized by a failure to undergo comprehensive corneal wound repair through eight weeks (Figure 3). Within 24 h of exposure, full-thickness keratocytosis occurred. In all corneas, activated keratocytes (fibrocytes) accumulated at the wound periphery by one week, and invasion of fibrocytes and vascular endothelium into the central cornea occurred by three weeks. Resolving corneas exhibited full-thickness, comprehensive tissue repair, concomitant with a reduction in edema and the absence of corneal erosions (Figure S1). However, in MGK corneas, tissue repair was only partially successful, typically surrounding acellular regions. In these corneas, the refractory areas were highly edematous with stromal rarification and distortion, and fibrocytic invasion appeared to be misdirected by the aberrant stromal topology (e.g., Figure 3E). Regardless of outcomes, quiescent keratocytes were not observed within the exposed stroma through eight weeks.

### Ultrastructural Characterization Suggests MGK Onset Involves Progressive Corneal Degeneration

**Figure 4:**
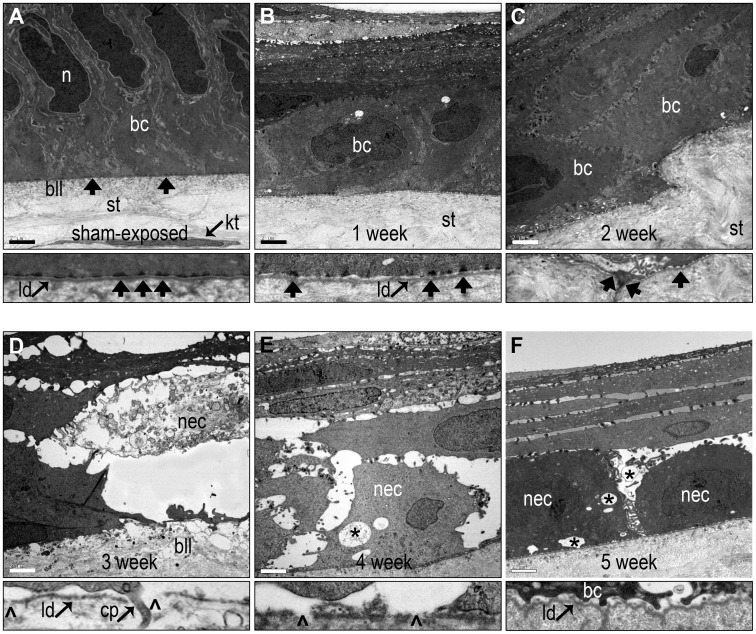
Changes in the ultrastructure of the BMZ from 1–5 weeks during the onset of MGK. (A–F) TEM images from control and 1–5 weeks. Scale bar  = 2 µm. Thin panels represent high magnification images of the BMZ. Basal cell (bc); hemidesmosomes (arrows); nucleus (n); stroma (st); Bowman’s like-layer (bll); necrotic basal cell (nec); proteinaceous void (*); lamina densa (ld); epithelial process penetrating into stroma (c); and breaks in the lamina densa (arrowheads).

Anticipating that the greater resolution of TEM would provide additional information about structural aspects of the chronic injury, we evaluated the fine structure of the CE and BMZ from 1–5 weeks after vapor exposure (Figure 4). We previously reported the regeneration of a partially stratified, differentiated CE by one week, with maturing basement membrane zone (BMZ) and rudimentary hemidesmosomes [11]. Reconstruction of the epithelium continued through two weeks, with abundant desmosomal and hemidesmosomal plaques, stratification into normally distributed intermediate, wing and superficial cells and an articulated tear film interface. Two structural abnormalities were regularly observed at two weeks in all corneas: focal swelling of the anterior stroma that was associated with destabilization of the overlying basal epithelial cells (Figure 4C); and scattered evidence of necrotic basal and suprabasal corneal epithelial cells (Figure S2A).

Despite ultrastructural evidence of regeneration of the corneal epithelium through two weeks, MGK corneas abruptly developed diverse corneal pathologies by three weeks (Figure 4). The widespread necrosis of basal and suprabasal corneal epithelial cells resulted in the formation of proteinaceous, edematous voids beneath a cap of superficial epithelium. The basal lamina was extensively disorganized with numerous breaks in the lamina densa often associated with the formation of bullous keratopathies. Basal epithelial cell processes were frequently observed penetrating through the basal lamina and projecting into the Bowman’s like-layer (BLL, Figure 4D and Figure S2B). Anterior stromal edema resulted in disorganization of collagen fibrils and lamellae. Heterophils were seen scattered throughout the exposure area which also showed a complete absence of viable keratocytes (Figure S3). Consistent with histopathology, the chronic corneal injury appeared to be progressive. For example, although BMZ degeneration was apparent at three weeks, it was significantly worse in five-week MGK corneas, with an edematous, necrotic epithelium; the loss of the lamina densa leaving the stratum basale in direct contact with stromal collagen; a severely disorganized BLL with numerous heterogeneities of unknown provenance and minimal evidence of hemidesmosomal anchoring plaques.

**Figure 5:**
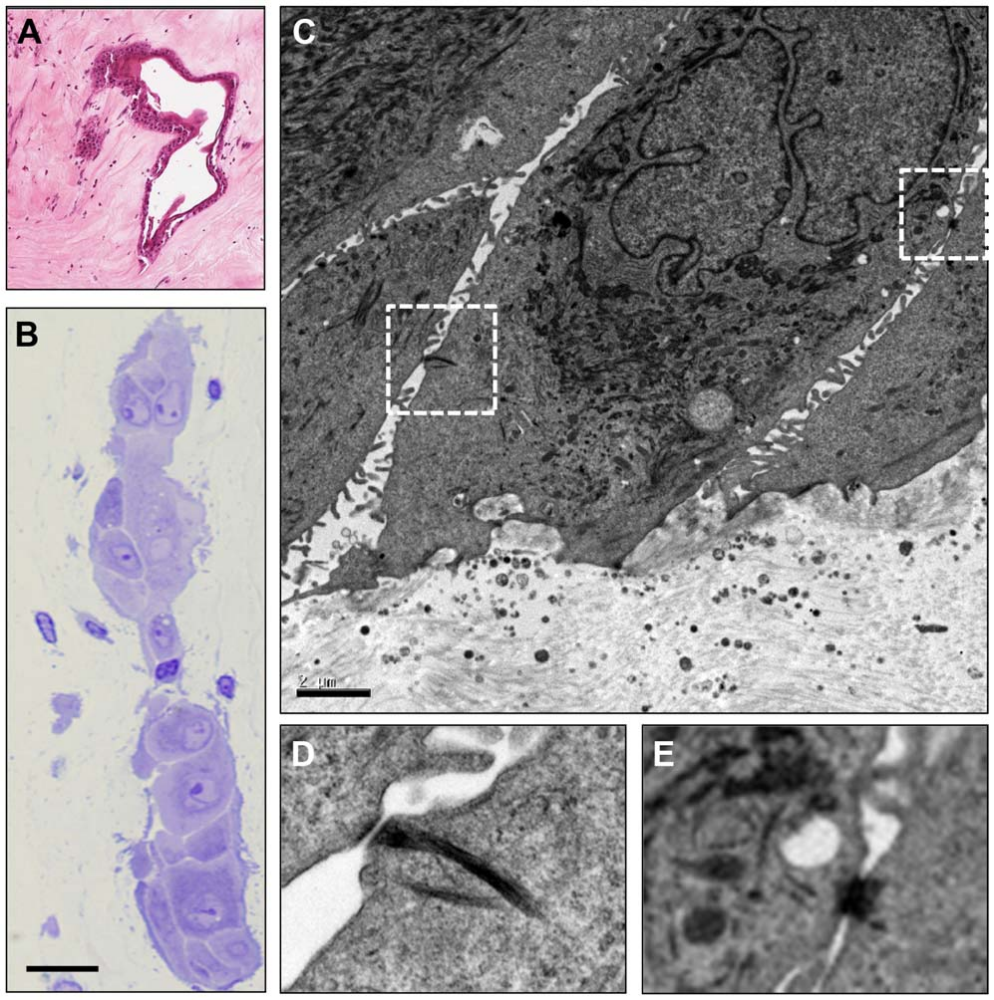
Intra-corneal deposition of epithelial cells during MGK. (A, B) Light microscopy demonstrating stromal residency of vacuolated (A) and compact (A, B) epithelial cell clusters. Scale bar in (B) is 40 µm. (C) Transmission electron micrograph of mid-stromal island demonstrating characteristic epithelial cell features. Scale bar is 2 µm. (D, E) Regions marked in (C) are shown at higher magnification to demonstrate desmosomal plaques.

In both five- and eight-week MGK corneas intrastromal clusters of differentiated squamous cells ranging in size from small groupings to large islands were observed within the stroma (Figure 5). These intracorneal cell clusters exhibited morphological markers of CE cells, including desmosomes and interdigitated cell processes, but without any apparent continuity with the corneal epithelium. A similar phenomenon was not observed in resolving corneas.

### Accumulation of Tissue Remodeling Effectors at Both the Anterior and Posterior Margins

**Figure 6:**
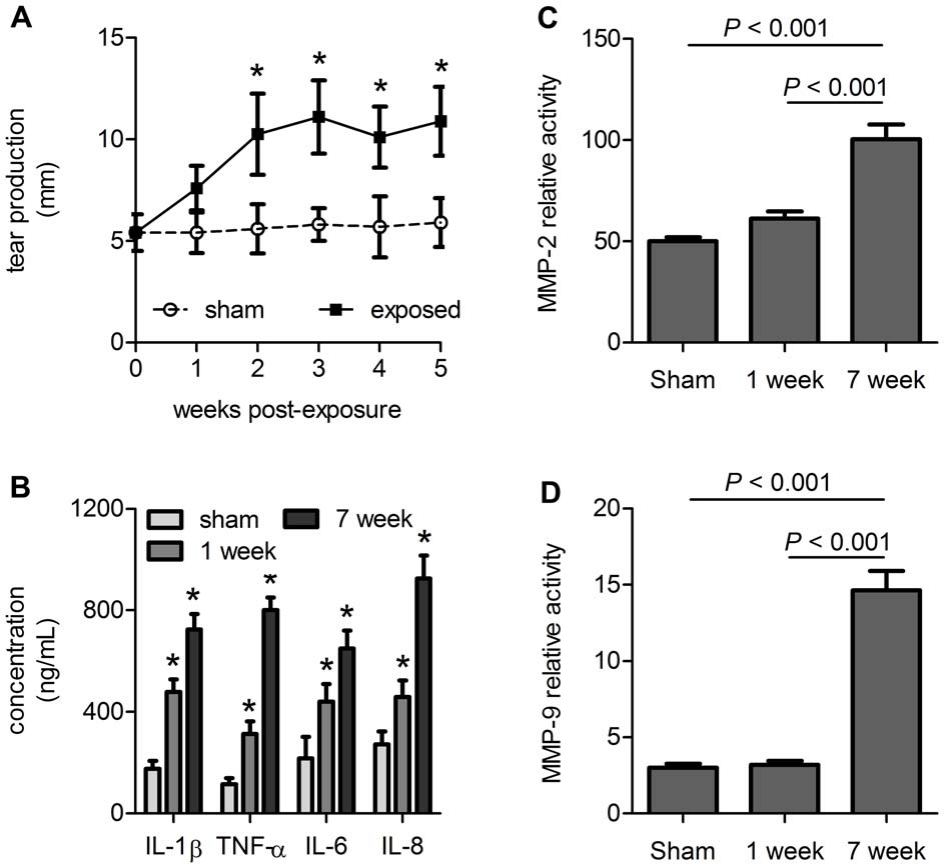
MGK corneas are exposed to keratoactive factors at the anterior and posterior boundaries. (A) Lacrimation increases over time in MGK corneas. (B) Cytokine levels in the AH increase during the acute and the chronic SM injury. For A and B: * represents *p*<0.05. (C, D) Measurement of (C) MMP-2 and (D) MMP-9 activity in the AH. All data are presented as mean plus standard error.

Since excessive corneal exposure to remodeling proteins and inflammatory mediators is a potential mechanism for structural degeneration, we evaluated the abundance of representative keratoactive proteins at the anterior and posterior corneal margins. Lacrimation doubled from 1–2 weeks and remained elevated through 5 weeks in MGK corneas (Figure 6A). Likewise, levels of four pro-inflammatory mediators (IL-1β, TNF-α, IL-6, and IL-8) were increased in the AH at one and seven weeks compared to matched, sham-exposed controls (Figure 6B). Levels of activated MMP-2 and -9 were also elevated in MGK corneas at seven weeks versus control corneas (Figure 6C and D). Significant activation was not observed at one week when the cornea was still recovering from the acute lesion. No corresponding changes in MMP activity or inflammatory mediators were observed in matched plasma samples (not shown), suggesting these changes were specific to the corneal injury.

## Discussion

### Structural and Biochemical Markers of MGK

A better understanding of the temporal changes that occur during the biphasic SM injury is crucial to mechanistic understanding and therapeutic development. To gain insight into the chronic form of corneal SM injury, we characterized exposed corneas from 1–8 weeks using histopathology and ultrastructural pathology. Consistent with clinical findings, we observed a biphasic response involving a transient improvement in corneal structure through two weeks, followed by the abrupt appearance and progressive deterioration of structure through eight weeks. The chronic injury appears fundamentally distinct from the acute lesion, representing injury mechanisms that operate on different time scales and in different corneal tissues. In addition to edema, preclinical structural abnormalities that may contribute to MGK onset were observed, including scattered basal epithelial cell necrosis and topological distortions in the BMZ with overlying epithelial destabilization. A histopathological comparison between MGK and resolved corneas at eight weeks suggested that MGK was associated with frustrated wound repair. MGK corneas also exhibited increased lacrimation and pro-inflammatory mediators and MMPs in the aqueous humor.

### Corneal Edema and the Chronic Phase of MGK Injury

Within days of exposure, edema causes corneal thickness to increase by over three-fold due, at least partially, to the entry of tear film into the stroma through the epithelial lesion [11]. Resolving corneas then undergo a progressive decrease in edema to baseline values over five weeks. In comparison, persistent edema is the leading clinical indicator of MGK development. Early administration of anti-inflammatories to suppress edema also delays the appearance of MGK symptoms, suggesting that stromal edema may be causative for a subset of MGK sequelae [9], [14]. Given the early appearance of stromal edema and the correlation between a decrease in stromal edema and injury resolution, we believe that corneal edema is the most significant pathology of the chronic SM injury. Comprehensive therapeutic treatments that directly address the cause of edema may therefore prevent or mitigate chronic sequelae.

In other stromal diseases, chronic edema evokes secondary keratopathies similar to those seen during MGK, including lamellar distortion, ocular haze, collagenolysis, neovascularization and epithelial bullae [17], [18]. Topologic stress may also impact corneal structure or cell function, e.g., by disrupting the adhesive contacts between corneal cells and extracellular matrices. A possible example of this can be observed in Figure 3B, in which uneven stromal swelling at the basement membrane is associated with destabilization of the overlying basal corneal epithelial cells.

Stromal edema can develop through dysfunction of the CE, corneal endothelium, limbus or leaky vasculature. Fluorescein uptake in MGK corneas and degeneration of the basement membrane zone indicate that CE disruption contributes to corneal edema. MGK corneas often exhibit relatively normal corneal thicknesses at the periphery, suggesting that lateral fluid transfer from the limbus is not likely to contribute significantly to edema (Figure 1). Fluid transfer from leaky neovasculature also is not likely to be an early contributor since central corneal neovascularization does not become apparent until after five weeks. Although technical limitations prevented us from evaluating structural evidence of endothelial decompensation, the fact that radiolabeled SM adducts develop on the lens after 5 min of vapor exposure raises the possibility that corneal endothelium may be directly exposed at sufficiently high doses [19].

### MGK Corneas Exhibit Delayed Stromal Wound Healing Processes

Complex autocrine and paracrine interactions between the corneal epithelium and stromal keratocytes are important in achieving the appropriate responses to corneal wound healing. Debridement of corneal epithelium from the lamina densa causes superficial keratocytes to undergo apoptosis and generates simple replacement within days by triggering mitosis and migration in adjacent keratocytes [20], [21], [22]. However, if epithelial loss includes an ulcerative injury, then release and penetration of TGF-β family members into the stroma activates peripheral keratocytes to become fibrocytes that will migrate to the site of injury and initiate repair processes [21], [22], [23]. These responses facilitate regeneration of a complete basement membrane and stromal remodeling.

SM exposure is distinct from most surgical and chemical injuries in that it combines epithelial vesication with full-thickness keratocytosis, requiring recellularization from the corneal periphery. Within a week of SM exposure, keratocyte activation was apparent at the wound margin, and within three weeks, centripetal migration of fibrocytes and vascular cells had begun. When comprehensive, full-thickness corneal repair was observed, stromal histopathology scores at eight weeks were dramatically improved versus MGK corneas, and slightly improved compared to two weeks. Alternatively, in MGK corneal repair appeared frustrated, with edematous stromal regions refractory to fibrocyte infiltration and deteriorated histopathology scores. Several mechanisms may underlay the inability of fibrocytes to penetrate the entire wound region, including altered chemokine signaling due to significant increases in stromal volume; disruption of the stromal matrix due to edema or enzymatic activity; pathological assembly of collagen matrices; or chemical derivatization of collagen by SM [24].

### MGK Involves Disruption of Epithelial Integrity

A fully stratified epithelium that excludes fluorescein is present in all corneas by two weeks after exposure (these data and [11]). MGK corneas subsequently begin to retain fluorescein again starting at three weeks [11]. Although it was initially believed that these lesions represented the loss of extensive regions of epithelium, gross epithelial lesions were not observed by histology. TEM confirmed that fluorescein retention occurs through focal disruptions in the integrity of the corneal epithelium with the demonstration of necrotic basal epithelial cells, destabilized CE and localized epithelial edema, but no instances of gross epithelial detachment except in cases of epithelial bullae.

Ultrastructural characterization revealed scattered evidence of basal cell necrosis (BCN) at two weeks after exposure and beyond in MGK corneas, but not in resolved corneas. The origin of this persistent BCN is unclear, but appears specific to SM exposure since other means of producing large scale epithelial loss, such as corneal scraping or excimer laser debridement, have not been associated with BCN [25]. The temporal delay between the exposure and the appearance of BCN between one and two weeks suggests that necrosis is either due to delayed SM toxicity or a second-order effect indirectly induced by SM exposure, and elucidating the mechanism(s) underlying this delay is crucial to therapeutic development. The development of widespread necrosis in the stratum basale is likely to have serious implications to corneal integrity. For example, necrosing epithelial cells release keratoactive proteins, such as pro-inflammatory mediators, matrix metalloproteinases and other signaling effectors that can activate receptor-mediated pathways in proximal cells. Basal epithelial cell necrosis structurally disrupts overlying and adjacent cells, and unless those cells are replaced will result in epithelial attenuation and a loss in differentiative capacity to regenerate the CE.

Fluorescein uptake in MGK corneas appears to be restricted to the area of the acute lesion, suggesting the necrotic mechanism may be bounded by some aspect of the exposure or recovery [11], [12]. It has been hypothesized that injury to the limbal stem cell (LSC) niche results in chronic CE cell loss, similar to LSC deficiency [26]. However, the LSC hypothesis is complicated by the lack of a direct injury to the limbus and the apparent restriction of BCN within the exposure margins. Other possibilities include delayed toxicity of basal CE cells that were originally located at the periphery of the exposure (e.g., genotoxicity of the transient amplifying population); destabilizing chemical modification of the basement membrane; or cell death in response to aberrant interactions with other corneal or adnexal tissues. Many of these mechanisms appear incongruent with decade-long asymptomatic periods in humans (assuming that the delayed form of MGK involves the same mechanism as chronic MGK). An alternative explanation is that basal cell necrosis is secondary to chronic stromal edema, resulting from dysfunction of another corneal barrier [17], [18].

### Degeneration of the Basement Membrane

Progressive degeneration of the basal lamina was observed during the development of MGK, involving breaks in the lamina densa, distortion of the BLL related to edema, loss of hemidesmosome plaques and redundant deposition of lamina densa. The corneal epithelium acts as a barrier between the tear film and the stroma. Disruption of the stratum basale and/or basal lamina permits transfer of keratoactive substances such as pro-inflammatory mediators and remodeling proteins from the epithelial compartment into the stroma and vice-versa through nano-scale pores in the basement membrane [27]. The introduction of pro-inflammatory mediators into this abnormal signaling environment by necrosing epithelial cells, infiltrating immune cells, neovascularization and through the limbus is likely to further contribute to corneal degeneration [28].

The correlation between overt gaps in the basal lamina and proteinaceous remnants of focal BCN is intriguing (e.g., Figure 4). One possibility is that CE necrosis is causally downstream of dissolution of the underlying lamina, for example in response to subepithelial edematous accumulation. Alternatively, basal necrosis may release active matrix proteins that disrupt the underlying basal lamina, such as MMPs -9 and -2. Whether the MMPs in the AH derive from corneal or adnexal tissues is unknown, but corneal entry due to anterior or posterior boundary dysfunction will further disrupt the stroma and basement membrane. MMPs play a major role in corneal remodeling and have been shown to be modulated in the tear film after ocular SM exposure; additionally, treatment of corneas with the MMP-9 inhibitor doxycycline slows MGK onset [9], [29], [30]. A causal relationship between release of MMP-9 from the corneal epithelium and basement membrane disruption has also been demonstrated [31], [32]. In these studies, corneal epithelial processes penetrated into the underlying stroma through regions of dissolved basement membrane, similar to what is observed, implicating MMP activity in disruption of the basal lamina and providing another mechanism by which abnormal interactions between the epithelial and stromal compartments could exacerbate the chronic injury.

### Intracorneal Epithelial Cells

Although this is the first report of the intra-stromal accumulation of epithelial cells following ocular SM exposure, similar phenomena have been observed in humans following surgical interventions that elicit severe corneal edema, such as deep excimer annular keratectomy [33]. The provenance of SM-induced intra-corneal epithelial cells is unknown, but may result from several factors, including a mechanically weakened BM, profound corneal edema, and wound healing processes in the anterior stroma. These islands may result in corneal pathologies through disruption of light refraction or induction of stromal degradation through the release of disruptive signaling mediators [21], [34], [35], [36]. In this study, epithelial islands had no apparent continuity with the corneal surface epithelium and are therefore distinct from epithelial ingrowth beneath corneal flaps (such as can occur following laser-assisted *in situ* keratomileusis) [37].

TEM and H&E data suggest two potential mechanisms for the stromal accumulation of CE cells. The first is projection of basal epithelial cells into the anterior stroma through breaks in the basement membrane (Figure 4 and Figure S2). Assuming complete penetration into the stroma, isolated epithelial cells may then aggregate and possibly divide. The second mechanism involves the entrapment of a segment of CE by stromal expansion during profound edema. The growth of new basal epithelial cells over the junction during cycles of epithelial separation and regeneration could therefore form an infolded epithelial pocket containing a central lumen. Whether these islands are clinically significant in the short- or long-term is unclear, as is whether improved control over cornea edema would prevent their development.

It is important to determine whether this phenomenon occurs in human cornea or other dermal tissues exposed to SM. If so, an improved understanding of the mechanism by which epithelial cells become localized within the stroma and the implications of these islands to wound repair and recovery is essential.

### Summary

While the acute ocular SM injury appears to share many commonalities with cutaneous exposures, the progression and extent of chronic SM lesions in ocular tissue are significantly more severe than in skin. The standard of care for acute SM lesions is similar to that of other corneal injuries (e.g., irrigation, antibiotics and steroids); however, these do not prevent MGK [2], [8], [38]. Corneal SM exposure simultaneously involves an acute epithelial necrosis and full-thickness keratocytosis, which in some cases transitions to a chronic injury phase characterized by persistently elevated edema and corneal degeneration through at least eight weeks after exposure. Here we show that the chronic injury is fundamentally distinct from the acute lesion, representing injury mechanisms that operate on different time scales and in different corneal tissues. MGK onset is associated with sequelae distinct from the acute injury, including basal epithelial cell necrosis, degeneration of the basement membrane and frustrated corneal wound repair. These structural pathologies are likely to contribute to delayed clinical sequelae such as neovascularization, persistent corneal lesions and increased corneal edema. Consequently, we have identified the ongoing necrosis of basal and suprabasal CE cells and the degeneration of the BLL and basal lamina as two early structural sequelae that may be important to the persistent edema and wound repair delays associated with the development of MGK. Although this effort focused on structural changes within the anterior cornea that are associated with MGK onset, observations of persistent edema suggest that chronic SM injury could also involve corneal endothelial dysfunction or limbal dystrophies.

## Supporting Information

Figure S1 **Visualization of resolved versus MGK corneas.** (A–D) Bright field and fluorescein images of resolved and MGK corneas at 8 weeks. (E) Corneal thicknesses of MGK, resolving and sham-exposed corneas over 8 weeks. Data are presented as mean plus standard error.
(TIF)

Figure S2 **Transmission electron micrographs demonstrate BMZ disruption, stromal distortions and disorganized BLL as early as 2 weeks.** (A) Cellular debris from a recently necrosed basal cell within the stratum basale, beneath which a pseudopod from a proximal basal corneal epithelial cell is re-epithelializing the denuded surface. Scale bar is 1 µm. (B) Example of a corneal epithelial process penetrating through the lamina densa into the stroma at 3 weeks. Scale bar is 200 nm in all panels. Basal cell (bc); cell process (cp); Bowman’s-like layer (bll); lamina densa (ld); necrotic debris (nec).
(TIF)

Figure S3 **Transmission electron micrographs of mid-stromal architecture demonstrates persistent stromal rarification and distortion, keratocytosis and inflammatory infiltrates in MGK corneas.** (A) 2 weeks; (B) 3 weeks; (C) 5 weeks and (D) 8 weeks. Scale bar is 2 µm in all panels. Necrotic keratocyte (nk); heterophil (hp); stromal edema (*).
(TIF)
