# Supplementary figures and images for: Architectural and Biochemical Expressions of Mustard Gas Keratopathy: Preclinical Indicators and Pathogenic Mechanisms
Source: PLoS One. 2012 Aug 10;7(8):e42837. doi: 10.1371/journal.pone.0042837 (PMC3416783; doi:10.1371/journal.pone.0042837)

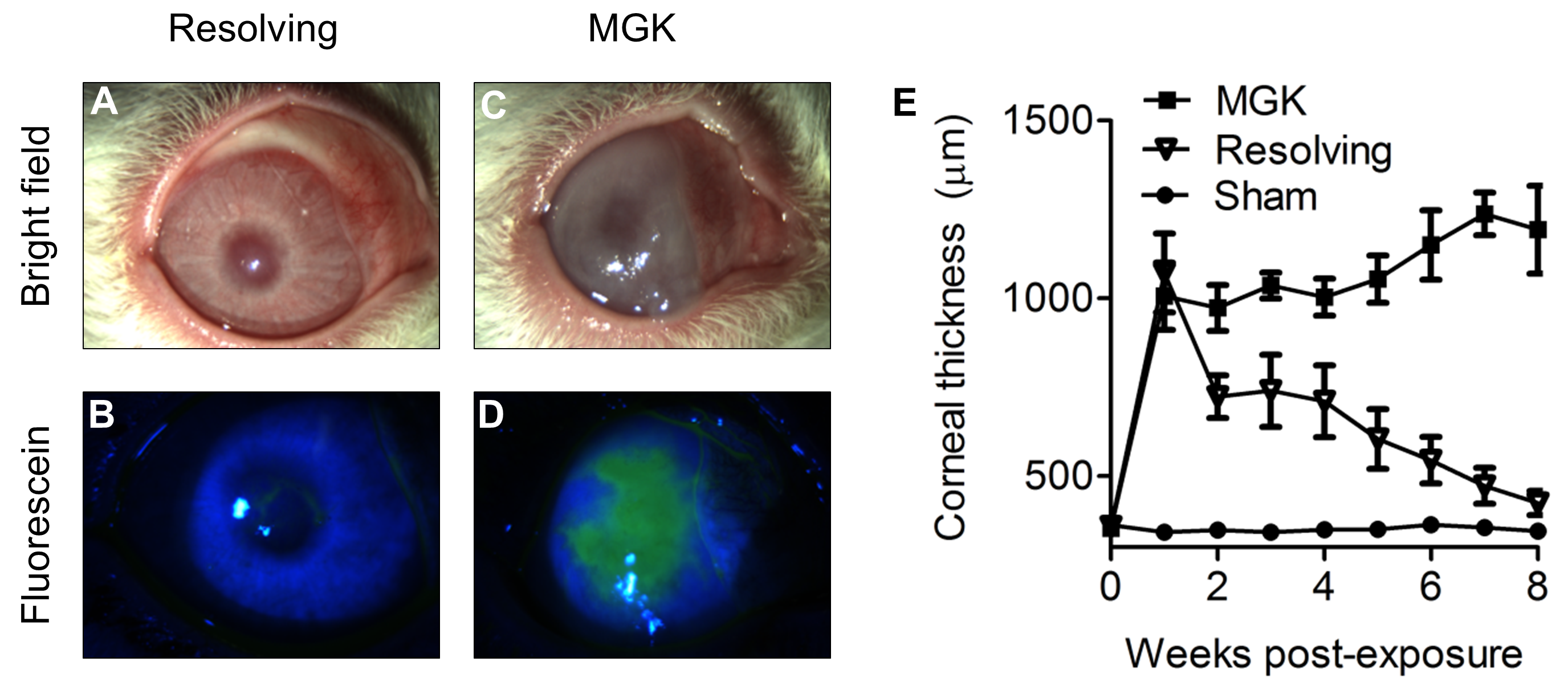

Supplement: Figure S1 — Visualization of resolved versus MGK corneas. (A–D) Bright field and fluorescein images of resolved and MGK corneas at 8 weeks. (E) Corneal thicknesses of MGK, resolving and sham-exposed corneas over 8 weeks. Data are presented as mean plus standard error. (TIF) [file pone.0042837.s001.tif]

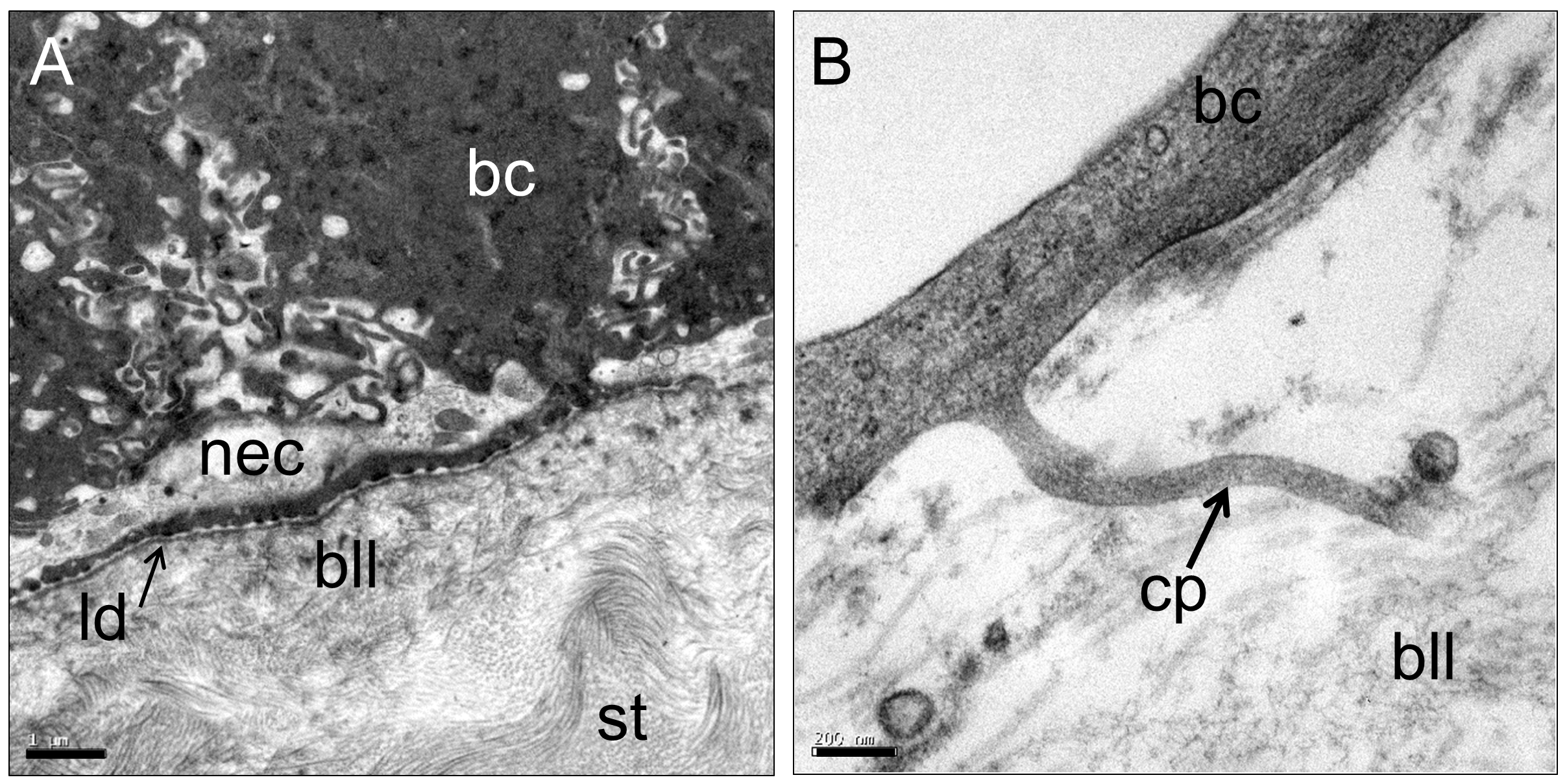

Supplement: Figure S2 — Transmission electron micrographs demonstrate BMZ disruption, stromal distortions and disorganized BLL as early as 2 weeks. (A) Cellular debris from a recently necrosed basal cell within the stratum basale, beneath which a pseudopod from a proximal basal corneal epithelial cell is re-epithelializing the denuded surface. Scale bar is 1 µm. (B) Example of a corneal epithelial process penetrating through the lamina densa into the stroma at 3 weeks. Scale bar is 200 nm in all panels. Basal cell (bc); cell process (cp); Bowman’s-like layer (bll); lamina densa (ld); necrotic debris (nec). (TIF) [file pone.0042837.s002.tif]

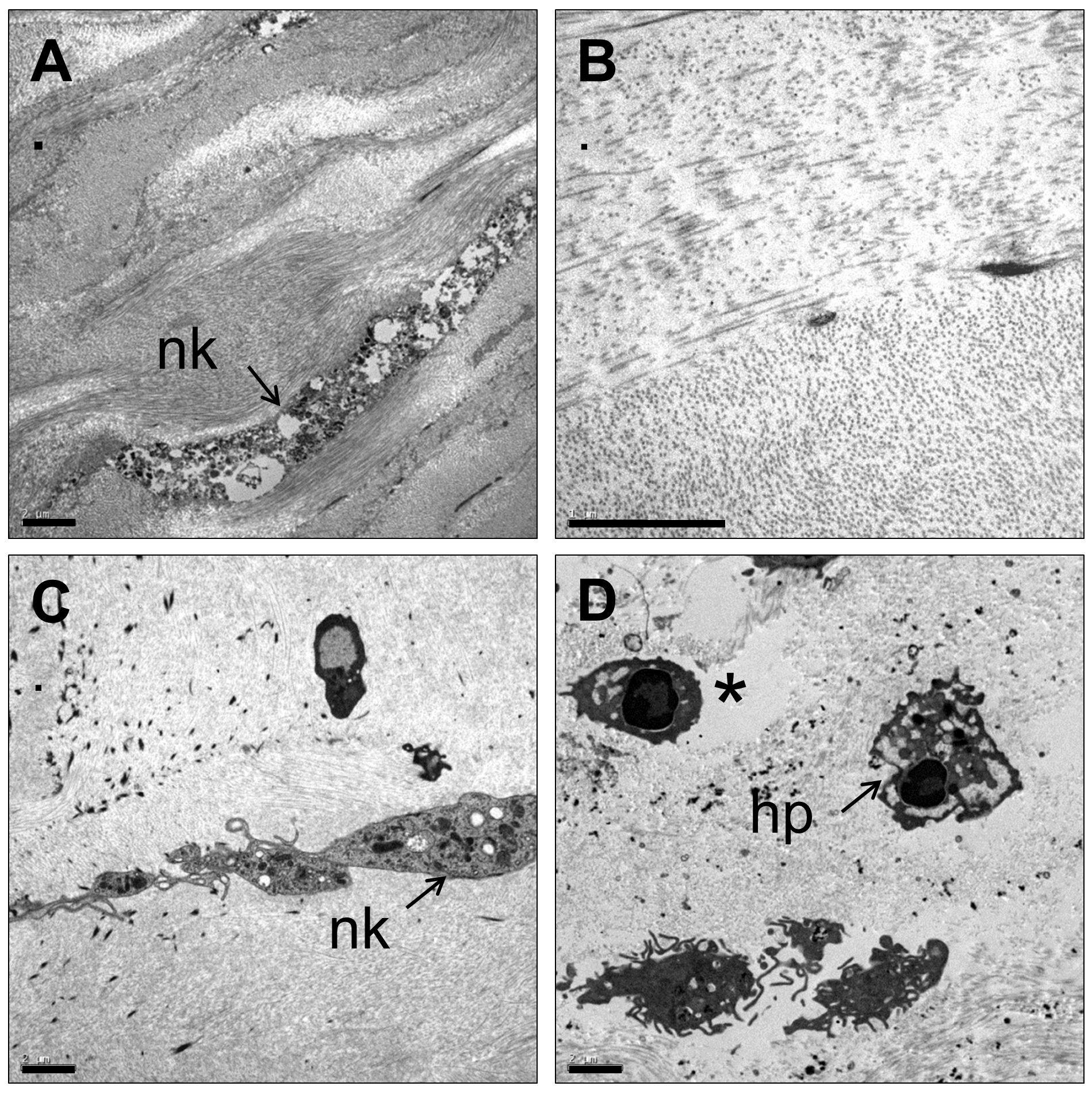

Supplement: Figure S3 — Transmission electron micrographs of mid-stromal architecture demonstrates persistent stromal rarification and distortion, keratocytosis and inflammatory infiltrates in MGK corneas. (A) 2 weeks; (B) 3 weeks; (C) 5 weeks and (D) 8 weeks. Scale bar is 2 µm in all panels. Necrotic keratocyte (nk); heterophil (hp); stromal edema (*). (TIF) [file pone.0042837.s003.tif]
